# Evaluation of ultrasound sensors for transcranial photoacoustic sensing and imaging

**DOI:** 10.1016/j.pacs.2023.100556

**Published:** 2023-09-17

**Authors:** Thomas Kirchner, Claus Villringer, Jan Laufer

**Affiliations:** a Institut für Physik, Martin-Luther-Universität Halle-Wittenberg, Von-Danckelmann-Platz 3, 06120 Halle (Saale), Germany; b Technische Hochschule Wildau, Hochschulring 1, 15745 Wildau, Germany

**Keywords:** Transcranial, Ultrasound sensors, Photoacoustic, Optoacoustic

## Abstract

Photoacoustic imaging through skull bone causes strong attenuation and distortion of the acoustic wavefront, which diminishes image contrast and resolution. As a result, transcranial photoacoustic measurements in humans have been challenging to demonstrate. In this study, we investigated the acoustic transmission through the human skull to design an ultrasound sensor suitable for transcranial PA imaging and sensing. We measured the frequency dependent losses of human cranial bones *ex vivo*, compared the performance of a range of piezoelectric and optical ultrasound sensors, and imaged skull phantoms using a PA tomograph based on a planar Fabry–Perot sensor. All transcranial photoacoustic measurements show the typical effects of frequency and thickness dependent attenuation and aberration associated with acoustic propagation through bone. The performance of plano-concave optical resonator ultrasound sensors was found to be highly suitable for transcranial photoacoustic measurements.

## 1 Introduction

Biomedical photoacoustic (PA) imaging and monitoring of the brain through the intact skin and skull has been challenging to demonstrate because cranial bone tissue causes strong acoustic attenuation and wavefront distortion [1], resulting in reduced image resolution and contrast [2].

Previous studies of PA wave propagation through cranial bone often relied on numerical simulations where the spatial distribution of the speed of sound and the mass density were estimated from X-ray computer tomography (CT) data. This typically involved the calculation of a porosity map [3] from clinical resolution X-ray CT in Hounsfield units or by defining a simplified layered skull model [4]. However, the limited spatial resolution of clinical CT images does not allow the representation of fine internal microstructure in acoustic propagation models [5] and introduces partial volume effects. Despite these limitations, conventional X-ray CT image data has been used to correct for wavefront aberrations in PA image reconstruction algorithms [2] or applications of clinical transcranial high intensity focused ultrasound (HIFU) [6]. More accurate simulations of transcranial acoustic wave propagation were demonstrated recently using high resolution X-ray micro-CT images [7].

PA measurements on the human brain *in vivo* through the intact skin and skull would lead to clinical applications such as monitoring of brain hemodynamics, the detection of intracranial bleeding and stroke diagnosis. The feasibility of PA brain monitoring was demonstrated experimentally *in vivo* on small [8], [9], [10] and large animals [11], [12], [13], and on humans who had undergone hemicraniectomy [14]. While transcranial PA imaging has been investigated in tissue-mimicking phantoms [15], and using *ex vivo* skulls of small animals [16], larger primates [1] and humans [2], transcranial PA measurements have yet to be demonstrated in humans *in vivo*. The main challenge lies in the acoustic attenuation and wavefront aberration caused by cranial bone tissue [4], [17]. These effects are highly frequency dependent [18] and increase with bone thickness and porosity. In addition, the initial PA pressure that can be generated in the brain is limited by the maximum permissible exposure [19] on skin and by the optical attenuation in tissue. Given that the optical attenuation of skull is similar to that of soft tissue, and should therefore result in sufficient optical penetration depth, the propagation of the PA waves through bone has a dominant effect on the signal-to-noise ratio.

It is therefore important to develop highly sensitive ultrasound (US) sensors that are optimised for this application. Previous studies have relied on piezoelectric sensors with large active element sizes as they are widely regarded as the most sensitive [20], [21] and are readily available. While highly sensitive optical US sensors have recently been reported [22], these technologies have not yet been employed for transcranial PA measurements.

In this paper, we investigate the feasibility of PA sensing and imaging of the human brain through the intact skull by focusing on the impact of the acoustic propagation through skull bone on PA images and the effect of the type of US sensor on PA detection sensitivity. We simulate the frequency dependent attenuation of broadband PA waves in human cranial bone and compare the *in silico* results with *ex vivo* transmission measurements made through skull bone using piezoelectric and optical US sensors. We fabricated and characterised plano-concave optical resonator (PCOR) sensors based on the design by Guggenheim et al. [22]. Our transcranial measurements showed that PCOR sensors offer greater acoustic sensitivity compared to large-area piezoelectric transducers, making them highly suited to transcranial PA measurements.

## 2 Materials and methods

### 2.1 Human skull sample

**Fig. 1:**
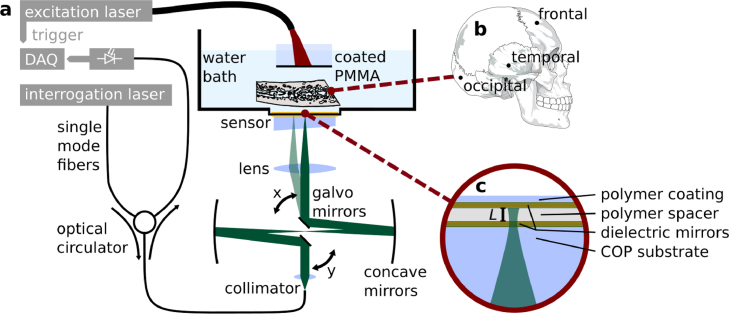
Fabry–Perot raster scanning setup for transcranial tomographic imaging of a planar PA source and a tube phantom. (a) Experimental setup for transmission measurements through *ex vivo* human skull. Target absorbers, such as a thin film of acrylic black paint on a PMMA substrate (shown here) or a tube filled with a liquid absorber, were illuminated by the output of an excitation laser and generated PA fields, these propagated through a region of cranial bone (see b) and were measured by scanning a interrogation laser spot across a planar Fabry–Perot sensor. (b) Diagram of a human skull and the measurement locations. (c) Schematic of the planar Fabry–Perot sensor.

The skull of a 70 year old male body donor was used for all measurements. Written informed consent for general scientific investigation was given by the body donor prior to death. A high resolution X-ray micro-CT scan of the skull and bone segmentation (see data set and descriptor [23]) provided the spatial distribution of the mechanical properties of bone and water for the numerical simulation of transcranial acoustic propagation. PA transmission measurements and PA imaging were performed across the temporal, occipital and frontal cranial bone as indicated in Fig. 1b. Skull bone is typically composed of an inner and an outer layer of solid cortical bone separated by a cancellous bone layer called diploë. Skull bone at these sites differs in thickness and porosity. The micro-structure is shown in supplemental figure 1. Temporal cranial bone is the thinnest (0.5–1.5 mm in the measured region) and is composed of the least cancellous tissue while frontal cranial bone is the thickest and most porous (6–9 mm in the measured region). The bone samples were immersed in degassed and deionised water at room temperature between 21 and 22 °C for at least 15 min before the measurements.

### 2.2 Simulation of transcranial acoustic propagation

3D PA wave propagation through frontal cranial bone was simulated using k-Wave [24]. We defined a two medium volume in a $364^{3}$ voxel grid, with 125 µm grid spacing, based on the bone segmentation obtained from the micro-CT data set [23] to represent the frontal cranial bone. The acoustic properties were set to those of bone with an acoustic attenuation α  =  2.7 dB/cm/MHz2, a mass density $\rho_{\text{bone}}$  =  2190 kg m^−3^ and a speed of sound $c_{\text{bone}}$  =  3100 m s^−1^, and those of water with a mass density $\rho_{\text{water}}$  =  1000 kg m^−3^, speed of sound $c_{\text{water}}$  =  1480 m s^−1^ and negligible acoustic attenuation. The PA source was represented by an initial pressure distribution in the shape of a disk of 5 mm diameter and 125 µm (single-voxel) height. The acoustic sensor was represented by single voxel positioned on the acoustic axis with the PA source at a distance of 3 cm (see also Fig. 3a). The frequency response of the simulated PA signals is limited by the computational grid (the maximum supported frequency is 6 MHz, the reference −6  dB bandwidth ranged from 0.5 MHz to 3.5 MHz, see *reference* in Fig. 3c). No frequency filters were used. Acoustic wave propagation was modeled in water as a reference and through frontal cranial bone in a water bath. The power density spectra of the signals were calculated using the Fourier transform, from which the bone insertion loss was obtained.

Only longitudinal pressure waves were modeled since shear waves can be neglected in the simulation of the acoustic transmission measurements on human skull. For the purposes of this study, shear waves are considered part of the overall acoustic attenuation. In cortical bone, shear waves are negligible at low angles of incidence (below 30°) [25] and although shear waves are generated in cancellous regions of the bone, they are attenuated strongly. Accounting for shear wave effects will be necessary for PA imaging with large sensor arrays where close-to-parallel incidence of PA waves on the skull is not ensured [2].

### 2.3 Transcranial PA imaging using a Fabry–Perot tomograph

To investigate the effects of transcranial acoustic wave propagation on PA images, simple absorbing structures were imaged though human cranial bone using a Fabry–Perot raster scanning tomograph (shown in Fig. 1a). The scanner is based on a previous design by Zhang et al. [26] and incorporates a planar Fabry–Perot (PFP) sensor described in Buchmann et al. [27]. The sensor consists of two dielectric mirrors (made from SiO_2_ and Ta${}_{2}$O${}_{5}$) separated by a parylene C spacer with a thickness of L  =  20 µm deposited on a cyclo-olefin polymer (COP) backing substrate as shown in Fig. 1c. The acoustic properties of the materials and the physical thickness of the sensor results in a broadband acoustic frequency response ranging from dc to 36 MHz (−3  dB).

The transduction mechanism of the sensor is based on the detection of acoustically induced changes in the reflected optical power of a cw interrogation beam following the pulsed excitation of PA signals in the target. The output of the interrogation laser (T100S, Yenista Optics, Lannion, France) was focused on the Fabry–Perot sensor. The beam waist had radius of 30 µm and the reflected intensity was coupled to an InGaAs photodiode (G9801-22, Hamamatsu Photonics K.K., Japan) using an optical circulator (CIR1550PM-APC, Thorlabs). The photodiode output was high-pass filtered at 50 kHz and recorded using a digital oscilloscope (PCI-5124, National Instruments, USA) at a sampling rate of 100 MS/s. 3D image data sets were acquired by raster scanning the interrogation beam across a detection aperture of 4 cm${}^{2}$ over a 216${}^{2}$-position raster using galvanometer mirrors (GVS012, Thorlabs) and recording the time-resolved PA signals at each position. A Nd:YAG excitation laser (Nano L 150–50, Litron Lasers, Rugby, UK) provided excitation pulses at 1064 nm with a duration of 7 to 9 ns and at a pulse repetition rate of 50 Hz, which also triggered the acquisition of PA signals at each scan point.

The water tank above the sensor was filled with degassed and deionised water in which the skull samples and PA sources were immersed. PA images of two absorbing targets were acquired. The first target consisted of a thin layer of black acrylic paint on a planar PMMA substrate. The layer of paint was illuminated directly by the divergent output of a multimode fiber. The pulse energy was 2 mJ and the spot size diameter was 5 mm, resulting in a planar PA wave with broadband acoustic frequency content. For the reference measurement without skull the excitation pulses were attenuated to 5% (0.1 mJ) using a neutral density filter. The PA source was positioned on the same acoustic axis as the sensor at a distance of 3 cm.

The second target mimicked the properties of a large blood vessel and consisted of a silicone tube (2 mm inner diameter) filled with a 2.2 M aqueous nickel sulfate solution (Sigma-Aldrich, CAS 10101-97-0) in a water bath. The tube was positioned at a distance of 2 cm from the sensor and was illuminated directly with 30 mJ pulses, resulting in a fluence of ≈  100 mJ/cm2. The absorption coefficient of the solution at 1064 nm was $\mu_{a}$  =  6 cm^−1^ and therefore comparable to the optical absorption of whole blood in the near infrared window [28]. The solution is likely to exhibit a higher Grüneisen coefficient compared to that of blood [29]. PA image data sets were acquired in water as a reference measurement and through skull bone by positioning either temporal, occipital, or frontal cranial bone between the PA source and the sensor. The PA image volumes are reconstructed using a fast Fourier transform algorithm [30] for a planar sensor using the k-Wave toolbox [24].

### 2.4 PA measurements of skull insertion loss with piezoelectric and optical US sensors

To investigate to what extent the type of US sensor affects transcranial PA measurements, the acoustic transmission through frontal cranial bone was measured using single element piezoelectric and optical sensors. Planar PA waves were generated as described above. Each US sensor was positioned at a distance of 3 cm parallel to the PA source and on the same acoustic axis. For each sensor, the insertion loss is calculated by dividing the Fourier transform of each waveform transmitted through the frontal bone by the transform of the reference measurement through the water bath.

Commercially available, large diameter piezoelectric immersion transducers with high acoustic sensitivity and low center frequency were used for the measurements. They included two unfocused lead zirconium titanate (PZT) transducers with an active element diameter of 25.4 mm, a bandwidth of 65% relative −6  dB at a center frequencies of either 500 kHz (V301-SU, Olympus, Waltham, USA) or 1 MHz (V302-SU, Olympus, Waltham, USA), and a broadband polyvinylidene fluoride (PVDF) sensor (Precision Acoustics Ltd, Dorchester, UK) with −6  dB at 20 MHz and an active element diameter of 19.2 mm. PA signals were recorded using a data acquisition unit with a sampling rate of 80 MHz (Flash ADC, PhotoSound, Houston, USA). 2500 waveforms were measured with each of the piezoelectric sensors.

Optical US sensors evaluated in this study include the PFP sensor used in the PA tomograph, the fabrication and characterisation of which was described by Buchmann et al. [27], and a set of plano-concave optical resonator (PCOR) sensors. PCOR sensors were chosen as they offer the lowest noise equivalent pressure (NEP) [22] of all optical PA sensors reported in the literature, and a broadband, non-resonant frequency response — attributes that are important for transcranial PA measurements. The fabrication and characterisation of the PCOR sensors is described in the following section. The largest PCOR sensor (L  =  493 µm) was used for the transcranial measurements. The PFP sensor (L  =  20 µm) was used to record 100 PA waveforms over a 1 mm2 grid where each waveform was averaged over 25 excitations. The optical sensors were interrogated with a beam of 30 µm waist radius.

### 2.5 Fabrication and characterisation of plano-concave optical resonator (PCOR) sensors

**Fig. 2:**
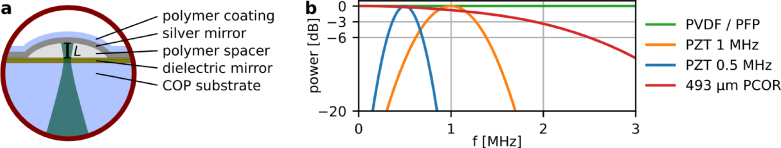
(a) Schematic of a plano-concave optical resonator (PCOR) sensor with a physical spacer thickness L. (b) Theoretical acoustic sensitivity spectra of a PCOR sensor [32] with L = 493 µm, two PZT-based, piezoelectric US transducers (frequency responses with a fractional bandwidth of 65%) and the ideal PFP and PVDF frequency response.

Fig. 2a shows a cross-section of the PCOR sensors. Planar substrates with a thickness of 9 mm were made using injection molding (Polymeroptix, Goch, Germany) of cyclo-olefin polymer (COP) (ZEONEX 480R, Zeon, Chiyoda, Japan). Dielectric mirrors consisting of SiO_2_ and TiO${}_{2}$ were deposited during two sputtering runs. The reflectivity of the mirrors was 94.9% and 97.8% at 1580 nm. Using an inkjet dispenser (AL 300, ficonTEC, Achim, Germany), droplets of a UV-curable liquid polymer (OrmoClad, Micro Resist Technology, Berlin, Germany) with low optical absorption in the 1530–1625 nm wavelength band were deposited on the first mirror. The droplets were varied in thickness (ranging from 100 to 500 µm) to provide sensors with different acoustic sensitivity and frequency response. The polymer was cured using ultraviolet (UV) light (UV-LED Solo P, Opsytec, Ettlingen, Germany) in a two-stage process. The droplets were illuminated immediately after deposition at a fluence of ≈  4 W/cm2 and a wavelength of 365 nm for 10 s. The substrates were then placed in a chamber flushed with pure nitrogen to counter the inhibitory effect of oxygen on UV curing [31] and again illuminated with UV light for at least 30 s. The second mirror consists of a 60 nm silver layer with a reflectivity of 97% and was deposited using DC magnetron sputtering (EM SCD 500 Leica, Wetzlar, Germany). A final protective barrier coating (10 µm, parylene C) was deposited to prevent water damage.

We characterised a total of ten PCOR sensors using NEP and metrics based on measured optical transfer functions, such as full-width half-maximum, fringe visibility, free spectral range, and Q-factor, and acoustic parameters, such as the cutoff frequency (or first minimum of the frequency response) and the −3 dB bandwidth.

The NEP [20] of the PCOR sensors fabricated in this work was measured using the method outlined in supplemental materials. The PCOR sensor with the lowest NEP was used to measure the insertion loss of frontal cranial bone. The theoretical frequency responses of the PZT and PVDF transducers used in this study and that of a PCOR sensor with a spacer thickness of L = 493 µm are shown in Fig. 2b. While resonant PZT transducers suffer from low sensitivity at low acoustic frequencies, PVDF and PCOR sensors have – at least in principle – a near uniform frequency response from dc to several MHz. The frequency response of the PCOR sensors in this work was modeled as described by Beard et al. [32]. To allow a qualitative comparison of the frequency responses of the sensors used in this work, broadband PA waveforms were generated in the planar absorber. The acquired PA signals were Fourier transformed to obtain power density spectra.

## 3 Results and discussion

### 3.1 Transcranial ultrasound propagation in silico

**Fig. 3:**
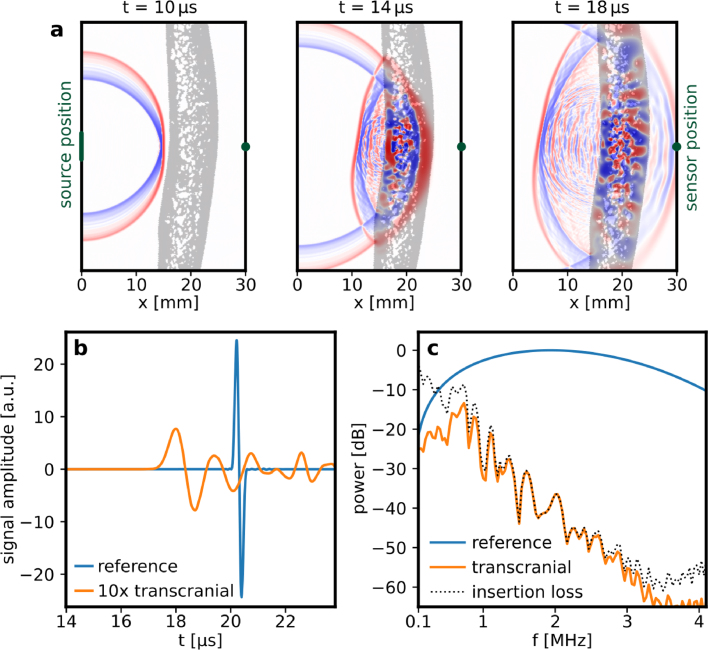
Acoustic propagation through frontal cranial bone *in silico*. (a) Snapshots of the center slice of a 3D acoustic field at three point in time. The PA wave originates from a 5 mm diameter disk PA source at x=0 and propagates across frontal cranial bone (for full series see supplementary video 1). The gray area represents skull tissue and was assigned the acoustic properties of bone. The green dot indicates the position of the US sensor. High pressure regions are illustrated in red, low in blue. (b) Single waveforms detected by the US sensor at a distance of x = 30 mm from the acoustic source. The waveform transmitted through water only is shown for reference, the waveform transmitted through skull is attenuated strongly and therefore displayed at a factor 10 magnification. (c) Acoustic power spectra of the detected waveforms and the calculated insertion loss.

The numerical simulation of US propagation through human frontal cranial bone is illustrated in Fig. 3a, which shows 2D snapshots of the acoustic field 10 µs, 14 µs and 18 µs after the generation of a PA wave. A video of the wave propagation is provided in supplementary video 1.

Fig. 3b shows the waveforms acquired by a point detector on the acoustic axis at a distance of x = 3 cm from the planar acoustic source. The reference signal (blue line) corresponds to acoustic propagation through water while the transcranial signal (orange line) shows the effects of strong attenuation and reverberations at later times in the signal that are the result of acoustic transmission through cranial bone. The attenuation is mainly caused by multiple reflections within cancellous bone due to acoustic impedance mismatch and to a lesser degree by acoustic attenuation within solid bone. This was verified by conducting additional simulations with attenuation coefficients α ranging from 2.7 to 6.0 dB/cm/MHz2. The change in the attenuation coefficient of solid bone was found to have a negligible effect on the overall attenuation.

Fig. 3c shows the frequency dependent insertion loss calculated from the power spectra of the transcranial and reference waveforms. It shows a reduction in the transmitted acoustic power by approximately −20 dB at 1 MHz. The insertion loss increases with frequency at a rate of around −10 dB/MHz. Fig. 3c indicates that PA measurements through the frontal cranial bone may be feasible at frequencies below 2 MHz.

### 3.2 Ex vivo transcranial PA tomography using a planar Fabry–Perot sensor

**Fig. 4:**
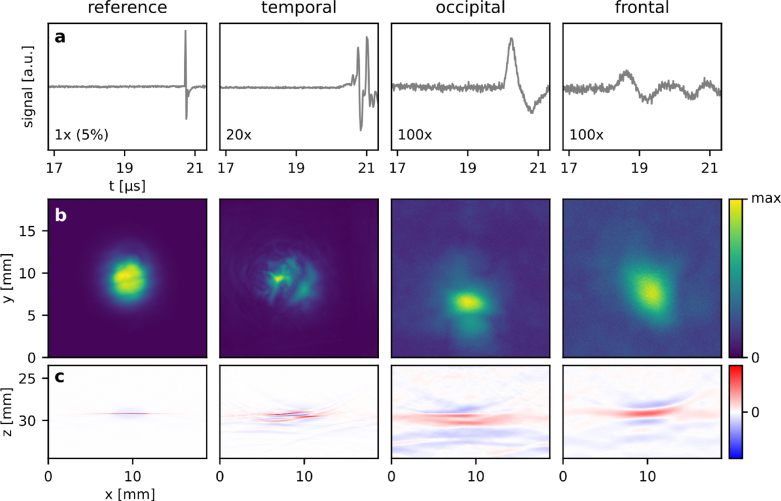
PA imaging of a layer of black paint on a PMMA substrate source using a planar Fabry–Perot sensor. The left column shows the reference measurements without skull, the columns to the right show PA measurements through temporal, occipital and frontal cranial bone. (a) Representative PA waveforms acquired at a single detection point. (b) *x–y* maximum intensity projections (MIP) of the reconstructed PA images shown in a linear color scale. (c) Central *x–z* slice shown in a diverging linear color scale.

Fig. 4 shows the image data sets of a planar absorber measured through water and temporal, occipital or frontal cranial bone. Representative PA waveforms measured at a single point through water (reference) and the different types of bone tissue are shown in Fig. 4a. As expected, the attenuation is lowest for the comparatively thin and least cancellous temporal bone while an attenuation of more than two orders of magnitude was observed in the thicker and more cancellous occipital and frontal cranial bone.

**Fig. 5:**
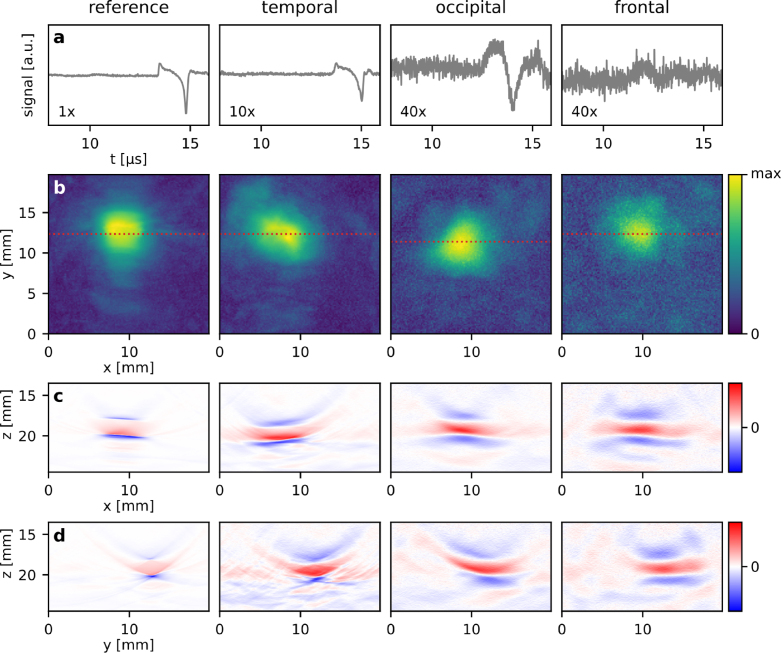
PA imaging of a tube with 2 mm inner diameter and filled with a 2.2 M NiSO${}_{4}$ solution using a planar Fabry–Perot sensor. The left column shows the reference measurement without the skull, the columns to the right show the measurements through temporal, occipital, and frontal cranial bone. (a) Representative PA waveforms acquired at a single detection point. (b) x–y MIPs of the reconstructed PA images shown in linear color scale. (c) Selected x–z longitudinal slices along the red dotted lines in (b) shown in a linear color scale. (d) Selected y–z axial slices at x = 9 mm.

Maximum intensity projections (MIP) of the reconstructed 3D images are shown in Fig. 4b and central slices in Fig. 4c. A comparison of the MIPs of the reference measurement and for temporal bone shows that the transmission of PA waves through a comparatively thin and least porous bone results in significant aberrations and reverberations as evidenced by the distorted visualisation of the planar PA source in the reconstructed images. The effects of wavefront aberration and frequency dependent attenuation are more significant in occipital and frontal cranial bone. While the location of the planar PA source can be discerned, its shape is distorted and smeared across a much larger volume. The resolution we observed in transcranial MIPs is on the order of 1 mm. It should be noted that we assumed a homogeneous speed of sound in the reconstruction algorithm. Its value was varied depending on bone thickness. For the images in Fig. 4, Fig. 5, the assumed speed of sound ranged from 1480 m s^−1^ for the reference measurement to 1660 m s^−1^ for frontal cranial bone. While more advanced algorithms have been reported that use additional information for a more accurate reconstruction, such as the distribution of acoustic properties inferred from X-ray CTs [2], the focus of this work is on the investigation of the relative merits of different types of US sensors. Also, coregistered X-ray Micro-CT data would not be available in a clinical setting. We therefore opted for a simple heuristic reconstruction as it illustrates the challenges in transcranial PA imaging.

The effects of wavefront aberration and attenuation can also be seen in the image data sets acquired in the vascular phantom (Fig. 5). While the amplitude of the PA signal excited in the absorber-filled tube is attenuated by an order of magnitude after propagating through temporal bone (Fig. 5a), its shape is in reasonable agreement with that of the reference. This suggests that its frequency content has not been strongly affected, which is confirmed by the MIPs and image slices of the reconstructed image volumes in Fig. 5c–d in which the image acquired through the temporal bone is, apart from minor distortions, comparable to the reference image. By contrast, the PA signals and images acquired through the thicker and porous occipital and frontal cranial bone tissue show strong attenuation and smearing of the image due to wavefront distortion. While the absorption coefficient of the NiSO${}_{4}$ solution is comparable to that of whole blood [28], the fluence used to excite PA waves is up to two orders of magnitudes higher than what we would optimistically expect to transmit into the brain in an *in vivo* imaging scenario given the maximum permissible exposure limits [19]. Despite the high fluence in the tube, the PA signal measured through frontal cortical bone was dominated by noise. The planar Fabry–Perot sensor employed in this study is therefore not well suited to PA transcranial brain imaging. This is not surprising since it is optimised for superficial, high resolution PA tomography. Its broadband frequency response from dc to tens of MHz is not well matched to the requirements of transcranial PA detection, which require high signal-to-noise ratios over a comparatively narrow frequency band from dc to 2 MHz as suggested by Fig. 3c.

Importantly, interferometric optical PA sensors can be designed to fulfill these requirements. Increasing the optical thickness of the resonator will not only reduce the frequency response of the sensor but also maximise its acoustic sensitivity — an approach we pursued by developing plano-concave optical resonator sensors.

**Fig. 6:**
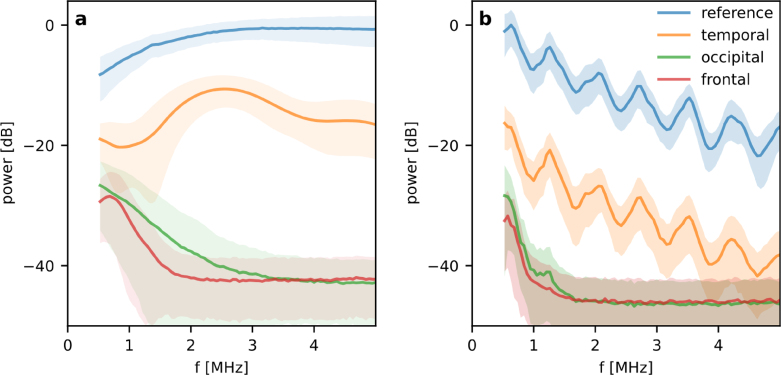
Acoustic power spectra of the image data sets measured in transcranial PA imaging experiments. The mean is shown as a solid line and the standard deviation is shown as a shaded region. To allow a meaningful comparison, the spectra were calculated from the top 5% of PA signals in terms of amplitude within each image data set. (a) Power spectra of a planar broadband PA source (thin black coating on a PMMA block), and (b) a vascular target (silicone tube with a 2 mm inner diameter, filled with a NiSO${}_{4}$ solution).

The thicker and more porous the bone tissue, the stronger the effects of acoustic attenuation and aberration, and therefore the loss of image resolution and blurring of features. The reduction in image resolution is caused by the frequency dependent attenuation of the bone tissue which is shown in Fig. 6a for the planar, broadband PA source. In the measurements through the temporal bone, higher acoustic frequencies are transmitted compared to the measurements made in occipital and frontal bone, which primarily transmit frequencies below 2 MHz. The temporal bone is also called the transcranial window and used clinically for Doppler-US measurements on a sub-cranial artery using transducers with a center frequency around 2 MHz. This trend is also confirmed by the measurements made in the tube phantom (Fig. 6b) albeit with a reference spectrum that is determined by the optical and material properties of the target itself.

### 3.3 Ex vivo transcranial PA measurements with low-frequency ultrasound sensors

While the PA imaging results in Fig. 4, Fig. 5, Fig. 6 suggest that transcranial PA measurements and imaging are feasible, the question remains what effect the choice of US sensor has on the sensitivity of the measurement. Given that bone strongly attenuates acoustic frequencies above 1.5 MHz, an intuitive choice would be to select piezoelectric transducers with a large active area to maximise acoustic sensitivity. In this section, low-frequency piezoelectric and optical US sensors are used for PA transmission measurements through frontal cranial bone.

**Fig. 7:**
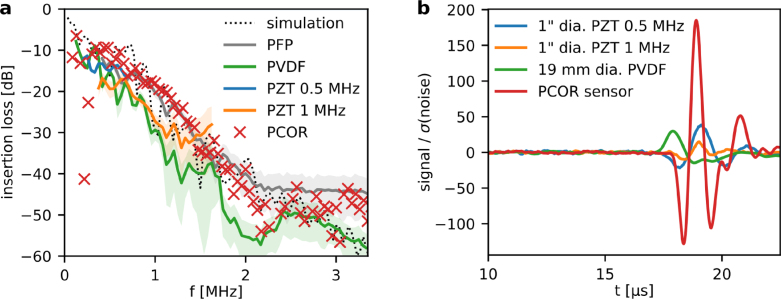
Frequency spectra and PA waveforms measured through frontal cranial bone using piezoelectric and optical sensors: Two PZT transducers with 25.4 mm active element diameter and center frequencies of 0.5 MHz and 1.0 MHz, one broadband PVDF transducer with 19.2 mm active element diameter, a planar Fabry–Perot (PFP) sensor, and PCOR sensor with a thickness of L = 493 µm. (a) Mean (solid line) and standard deviation (shaded) of the insertion loss of the sensors. The results of the numerical simulation are shown for comparison (dotted line). (b) Representative PA waveforms measured through the frontal cranial bone using the sensors. All waveforms are bandpass filtered and normalised with respect to the detection noise.

The frequency dependent insertion loss of *ex vivo* frontal human cranial bone was measured using three large active area piezoelectric transducers and two optical sensors with comparatively small active element sizes, i.e., a planar Fabry–Perot sensor and a plano-concave optical resonator (PCOR) sensor. Fig. 7a shows the insertion loss measured in human frontal cranial bone. The insertion losses measured by the different US sensors are comparable and consistent, and are also in good agreement with the *in silico* results. The only noticeable difference arises due to the limited frequency responses of the two PZT transducers, which do not cover the full acoustic spectrum transmitted through frontal bone.

Fig. 7b shows representative PA waveforms measured using the large element size piezoelectric sensors and a PCOR sensor. All waveforms were band-pass filtered (second-order Butterworth filter, cut-on at 10 kHz, cut-off at 3 MHz) and normalised with respect to the standard deviation of the noise. Interestingly, the PCOR sensor clearly outperforms the three piezoelectric sensors despite orders of magnitudes difference in their active element size. We believe this is explained by a combination of three factors. First, the large active element size of the PZT and PVDF detectors may result in cancellation effects if heterogeneous acoustic fields, such as those transmitted through bone, are measured. The large size of the piezoelectric transducers available to us may therefore explain the low sensitivity to distorted wavefronts. Smaller, millimeter-scale PZT sensors may provide better sensitivity in this case but such sensors were not available for comparison. Second, the resonant frequency response of PZT transducers prevents the detection of some of the strongest frequency components of the transcranial PA field below 0.5 MHz, thus reducing the signal amplitude. Third, the inherent thermal noise of PZT materials is dependent on the element size, leading to increased noise in large detectors.

### 3.4 Optical characterisation, acoustic sensitivity and frequency response of PCOR sensors

Metrics derived from the measured optical transfer functions, i.e. full-width half-maximum, fringe visibility, free spectral range, and Q-factor are listed in supplementary table 1. The physical thickness L was calculated from the free spectral range of the sensors. Bandwidth and band-pass cut-off were estimated using a numerical model [32], the material parameters and L. The estimated values obtained using the model were in good agreement with measured data as evidenced by supplementary figure 2.

**Fig. 8:**
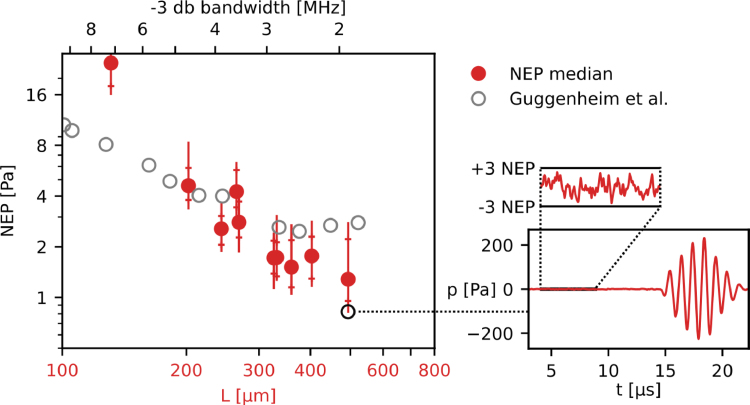
Noise equivalent pressure (NEP) of plano-concave optical resonator (PCOR) sensors as a function of sensor thickness and bandwidth. The sensitivity of PCOR sensors first reported by Guggenheim et al. [22] are shown for comparison. Note that the physical thickness L of the sensors refers to the PCOR sensors developed in this study. Median NEPs were calculated from 100 sequential measurements, the lines show minimum to maximum measured NEP with 5 to 95 percentile bars. The measurement which yielded the lowest NEP of 0.8 Pa is shown as an example.

Fig. 8 shows the NEP of the PCOR sensors as a function of thickness and the corresponding −3  dB bandwidth. Most sensors exhibit NEPs that are in broad agreement with those reported by Guggenheim et al.  [22]. The slightly lower NEP of the PCOR sensors made in this work may be explained by differences in the optical and mechanical properties of the materials, the structure of the PCOR sensors (e.g. a final encapsulating polymer layer was omitted), and the methods for measuring NEP. The thickest PCOR sensors reached NEPs of around 1 Pa (or $0.6\;\text{mPa}/\sqrt{\text{Hz}}$).

As shown in supplementary table 1, the NEP was measured at different output power settings of the interrogation laser (4 mW and 8 mW). The NEP did not improve linearly with interrogation laser power as initially expected. Given the high Q-factors of the PCOR sensors, this suggests that phase noise is playing a more dominant role at increased laser powers compared with laser intensity noise, thermal noise, and detection electronics noise.

PCOR sensors nevertheless offer promising opportunities for further improvements in acoustic sensitivity and frequency response. For example, the Q-factor may be improved by using dielectric mirrors of higher reflectivity as shown by Guggenheim et al.  [22]. In this work, the second mirror was made from silver as the lower temperature during the deposition process prevented thermal damage to the polymer spacer. This limited the mirror reflectivity and hence optical phase sensitivity. The omission of the final encapsulating polymer layer used by Guggenheim et al. may also result in acoustic reverberations within the dome-structure of the PCOR sensor. This may explain the deviations from the ideal frequency response (see supplemental figure 2) which manifest themselves as weak resonances at low frequencies. Further improvements in fringe visibility and optical phase sensitivity, and hence acoustic sensitivity, may also be achieved by matching the beam waist and divergence to the curvature and size of the PCOR [33].

We have shown in Section 3.3 that transcranial PA sensing through thick cranial bone requires US sensors with high sensitivity at frequencies below 1 MHz. While the frequency response of the largest PCOR sensors developed in this work (dc to 2 MHz) is well matched to the transmission spectrum of cranial bone, it is not completely uniform. This may be explained by internal acoustic reflections (see supplemental figure 2), which can be suppressed by applying an additional polymer layer to create acoustically homogeneous planar sensors [22]. Matching of the acoustic detection bandwidth with that required for optimal transcranial PA sensing could be achieved with PCORs twice the physical thickness of the largest sensors characterised here, which would yield further improvements in acoustic sensitivity.

The comparison of waveforms shown in Fig. 7b illustrates that PCOR sensors are better suited to transcranial PA measurements compared to conventional piezoceramic sensors with large element sizes. To achieve the NEP of PCOR sensors using previously reported piezocomposite transducers [21], an active element size roughly three orders of magnitude larger than that of our optical resonator is required. The intrinsic small element size of PCORs also results in less spatial averaging compared to large piezoelectric sensors.

## 4 Conclusions

We have shown both *in silico* and in PA transmission measurements through *ex vivo* human skull that ultrasound sensors with high acoustic sensitivity to low frequency ultrasound are required for transcranial PA measurements in humans. We have illustrated the effects of skull bone tissue on acoustic propagation and PA imaging. While the frequency dependent losses are dependent upon the type of skull bone, our evaluation of insertion losses has shown that frequencies below 1 MHz are least attenuated in occipital and frontal cranial bone while temporal bone exhibits a broader acoustic transmission spectrum. We designed, fabricated, and characterised an ultrasound sensor based on a plano-concave optical resonator, which combines advantageous attributes for transcranial PA measurements, such as high acoustic sensitivity, a broadband frequency response, and a small active element size radius of 30 µm.

## CRediT authorship contribution statement

**Thomas Kirchner:** Conceptualization, Data curation, Formal analysis, Investigation, Funding acquisition, Implementation, Validation, Visualization, Software, Writing – original draft, Methodology, Supervision, Project administration, Resources (also see non-author contributions in acknowledgements), Writing – review & editing. **Claus Villringer:** Methodology, Implementation (for PCOR sensor fabrication), Writing – review & editing. **Jan Laufer:** Methodology, Supervision, Project administration, Resources (also see non-author contributions in acknowledgements), Writing – review & editing.

## Declaration of competing interest

The authors declare that they have no known competing financial interests or personal relationships that could have appeared to influence the work reported in this paper.

## Data Availability

Raw PA and US data is available at doi:10.5281/zenodo.8163858 including the simulation code. The X-ray Micro-CT data set is available at doi:10.5281/zenodo.6108435. Data processing and analysis was primarily performed using the open-source k-Wave toolbox as described.

## Appendix A Supplementary data

Video S1 Acoustic propagation through frontal cranial bone in silico. A 3D k-Wave forward simulation of a flat acoustic source with 5 mm diameter. Time series of the pressure distribution in the center slice. The light gray area shows the skull segmentation used for the simulation.

MMC S1 Description of noise equivalent pressure (NEP) measurement, supplementary table 1 and supplementary figures 1–3.

## References

[1] Huang Chao, Nie Liming, Schoonover Robert W., Guo Zijian, Schirra Carsten O., Anastasio Mark A., Wang Lihong V. (2012). Aberration correction for transcranial photoacoustic tomography of primates employing adjunct image data. J. Biomed. Opt..

[2] Na Shuai, Yuan Xiaoyun, Lin Li, Isla Julio, Garrett David, Wang Lihong V. (2020). Transcranial photoacoustic computed tomography based on a layered back-projection method. Photoacoustics.

[3] Aubry J.-F., Tanter M., Pernot M., Thomas J.-L., Fink M. (2003). Experimental demonstration of noninvasive transskull adaptive focusing based on prior computed tomography scans. J. Acoust. Soc. Am..

[4] Liang Bingyang, Liang Bingyang, Wang Shaomeng, Shen Fei, Shen Fei, Liu Qing Huo, Gong Yubin, Gong Yubin, Yao Junjie, Yao Junjie (2021). Acoustic impact of the human skull on transcranial photoacoustic imaging. Biomed. Opt. Express.

[5] Pinton Gianmarco, Aubry Jean-Francois, Bossy Emmanuel, Muller Marie, Pernot Mathieu, Tanter Mickael (2012). Attenuation, scattering, and absorption of ultrasound in the skull bone. Med. Phys..

[6] Marquet F, Pernot M, Aubry J-F, Montaldo G, Marsac L, Tanter M, Fink M (2009). Non-invasive transcranial ultrasound therapy based on a 3D CT scan: Protocol validation and *in Vitro* results. Phys. Med. Biol..

[7] Robertson James, Urban Jillian, Stitzel Joel, Treeby Bradley E (2018). The effects of image homogenisation on simulated transcranial ultrasound propagation. Phys. Med. Biol..

[8] Wang Xueding, Pang Yongjiang, Ku Geng, Xie Xueyi, Stoica George, Wang Lihong V (2003). Noninvasive laser-induced photoacoustic tomography for structural and functional in vivo imaging of the brain. Nature Biotechnol..

[9] Wang Xueding, Xie Xueyi, Ku Geng, Wang Lihong V, Stoica George (2006). Noninvasive imaging of hemoglobin concentration and oxygenation in the rat brain using high-resolution photoacoustic tomography. J. Biomed. Opt..

[10] Kang Jeeun, Liu Xiuyun, Cao Suyi, Zeiler Steven R., Graham Ernest M., Boctor Emad M., Koehler Raymond C. (2022). Transcranial photoacoustic characterization of neurovascular physiology during early-stage photothrombotic stroke in neonatal pigletsin vivo. J. Neural Eng..

[11] Kirchner Thomas, Gröhl Janek, Holzwarth Niklas, Herrera Mildred A., Hernández-Aguilera Adrián, Santos Edgar, Maier-Hein Lena (2019). Photons Plus Ultrasound: Imaging and Sensing 2019.

[12] Kirchner Thomas, Gröhl Janek, Herrera Mildred A., Adler Tim, Hernández-Aguilera Adrián, Santos Edgar, Maier-Hein Lena (2019). Photoacoustics can image spreading depolarization deep in gyrencephalic brain. Sci. Rep..

[13] Petrova I.Y., Petrov Y.Y., Esenaliev R.O., Deyo D.J., Cicenaite I., Prough D.S. (2009). Noninvasive monitoring of cerebral blood oxygenation in ovine superior sagittal sinus with novel multi-wavelength optoacoustic system. Opt. Express.

[14] Na Shuai, Russin Jonathan J., Lin Li, Yuan Xiaoyun, Hu Peng, Jann Kay B., Yan Lirong, Maslov Konstantin, Shi Junhui, Wang Danny J., Liu Charles Y., Wang Lihong V. (2021). Massively parallel functional photoacoustic computed tomography of the human brain. Nat. Biomed. Eng..

[15] Poudel Joemini, Na Shuai, Wang Lihong V., Anastasio Mark A. (2020). Iterative image reconstruction in transcranial photoacoustic tomography based on the elastic wave equation. Phys. Med. Biol..

[16] Liang Bingyang, Liu Wei, Zhan Qiwei, Li Mucong, Zhuang Mingwei, Liu Qing H., Yao Junjie (2019). Impacts of the murine skull on high-frequency transcranial photoacoustic brain imaging. J. Biophotonics.

[17] Yao Junjie, Wang Lihong V. (2014). Photoacoustic brain imaging: From microscopic to macroscopic scales. Neurophotonics.

[18] Lee Jooho, Paeng Dong-Guk, Ha Kanglyeol (2020). Attenuation of the human skull at broadband frequencies by using a carbon nanotube composite photoacoustic transducer. J. Acoust. Soc. Am..

[19] ANSI (2007).

[20] Winkler Amy M., Maslov Konstantin, Wang Lihong V. (2013). Noise-equivalent sensitivity of photoacoustics. J. Biomed. Opt..

[21] Xia Wenfeng, Piras Daniele, van Hespen Johan C.G., van Veldhoven Spiridon, Prins Christian, van Leeuwen Ton G., Steenbergen Wiendelt, Manohar Srirang (2013). An optimized ultrasound detector for photoacoustic breast tomography. Med. Phys..

[22] Guggenheim James A., Li Jing, Allen Thomas J., Colchester Richard J., Noimark Sacha, Ogunlade Olumide, Parkin Ivan P., Papakonstantinou Ioannis, Desjardins Adrien E., Zhang Edward Z., Beard Paul C. (2017). Ultrasensitive plano-concave optical microresonators for ultrasound sensing. Nat. Photon..

[23] Kirchner Thomas (2022). http://arxiv.org/abs/2202.11519.

[24] Treeby Bradley E., Cox B.T. (2010). K-Wave: MATLAB toolbox for the simulation and reconstruction of photoacoustic wave fields. J. Biomed. Opt..

[25] White P.J., Clement G.T., Hynynen K. (2006). Longitudinal and shear mode ultrasound propagation in human skull bone. Ultrasound Med. Biol..

[26] Zhang Edward, Laufer Jan, Beard Paul (2008). Backward-mode multiwavelength photoacoustic scanner using a planar Fabry-Perot polymer film ultrasound sensor for high-resolution three-dimensional imaging of biological tissues. Appl. Opt..

[27] Buchmann Jens, Guggenheim James, Zhang Edward, Scharfenorth Chris, Spannekrebs Bastian, Villringer Claus, Laufer Jan (2017). Characterization and modeling of Fabry-Perot ultrasound sensors with hard dielectric mirrors for photoacoustic imaging. Appl. Opt..

[28] Jacques Steven L. (2013). Optical properties of biological tissues: A review. Phys. Med. Biol..

[29] Fonseca Martina B., An Lu, Cox Benjamin T. (2017). Sulfates as chromophores for multiwavelength photoacoustic imaging phantoms. J. Biomed. Opt..

[30] Köstli K.P., Frenz M., Bebie H., Weber H.P. (2001). Temporal backward projection of optoacoustic pressure transients using Fourier transform methods. Phys. Med. Biol..

[31] Alvankarian Jafar, Majlis Burhanuddin Yeop (2015). Exploiting the Oxygen Inhibitory Effect on UV Curing in Microfabrication: A Modified Lithography Technique. PLoS One.

[32] Beard P.C., Perennes F., Mills T.N. (1999). Transduction mechanisms of the Fabry-Perot polymer film sensing concept for wideband ultrasound detection. IEEE Trans. Ultrason. Ferroelectr. Freq. Control.

[33] Martin-Sanchez David, Li Jing, Zhang Edward Z., Beard Paul C., Guggenheim James A. (2023). ABCD transfer matrix model of Gaussian beam propagation in plano-concave optical microresonators. Opt. Express.

