# Supplementary material for: Evaluation of ultrasound sensors for transcranial photoacoustic sensing and imaging
Source: Photoacoustics. 2023 Sep 17;33:100556. doi: 10.1016/j.pacs.2023.100556 (PMC10658602; doi:10.1016/j.pacs.2023.100556)
Supplement: MMC S1 — Description of noise equivalent pressure (NEP) measurement, supplementary table 1 and supplementary figures 1–3. [file mmc2.pdf]

Supplemental Material – Evaluation of ultrasound sensors for transcranial photoacoustic sensing and imaging

Measurement of Noise Equivalent Pressure

Noise Equivalent Pressure (NEP) is defined as the pressure at which the peak positive signal amplitude, $\max(S)$, is equal to the standard deviation of the detection noise, $\sigma(\text{noise})$, i.e., NEP $= \sigma(\text{noise}) \cdot p_{\text{pp}}/\max(S)$, where p_{pp} is the peak positive pressure amplitude effected by a calibrated source. It can also be normalized over a specific detection bandwidth as NEP per $\sqrt{\text{bandwidth}}$.

The PCOR sensors were incorporated into the Fabry-Perot scanner described in section 2.3 using a Fabry-Perot tomograph. The sensors were operated using the same interrogation and data acquisition setup. The measurement of the acoustic sensitivity involved the use of a calibrated piezoelectric transducer (Sonotec, Halle, Germany) with an active element diameter of 20 mm and a center frequency of 1 MHz as an acoustic source. The transducer was placed in a water tank at a distance of 20 mm to the PCOR sensor and on the same acoustic axis. The sensor position within the sound field was chosen as it coincides with a local pressure maximum, thus minimising measurement bias toward underestimating NEP. The calibrated transducer was driven by a frequency generator (Agilent 33522A, Santa Clara, USA) to produce a tone burst consisting of a four period sine wave with 100 mVpp at a frequency of 1 MHz resulting in a peak positive pressure amplitude of $p_{\text{pp}} = 230\,\text{Pa}$ at the location of the PCOR sensor. $\sigma(\text{noise})$ was measured over 500 data points preceding the tone burst. The NEP of each PCOR sensor was estimated from the measured acoustic signal. To allow a comparison of

sensors of varying size and therefore acoustic detection bandwidths, a digital band-pass filter (2nd order Butterworth) was applied to the data. For each PCOR sensor, the pass-band ranged from 10 kHz to the first minimum in sensitivity (cf. supplementary figure 2 and listed as *cutoff frequency* in supplementary table 1). The NEP values correspond to the −3 dB bandwidth of each sensor, which was estimated using a numerical model by Beard et al. 1999.

Supplementary Video 1: Acoustic propagation through frontal cranial bone *in silico*. A 3D k-Wave forward simulation of a flat acoustic source with 5 mm diameter. Time series of the pressure distribution in the center slice. The light gray area shows the skull segmentation used for the simulation.

Supplementary Table 1: Parameters derived from the interferometer transfer function of ten PCOR sensors and the noise equivalent pressure for an interrogation laser power of 4 mW and 8 mW.

L [µm]	FWHM [pm]	visibility	Q	BP cutoff [MHz]	−3 dB BW [MHz]	med NEP 4 mW [Pa]	med NEP 8 mW [Pa]	min NEP 4 mW [Pa]	min NEP 8 mW [Pa]	min NEP(f) $[\frac{\text{mPa}}{\sqrt{\text{Hz}}}]$
493	27	90%	60000	4.0	1.9	1.38	1.28	0.85	0.82	0.60
403	30	53%	53000	4.9	2.3	2.59	1.76	1.80	1.17	0.77
360	21	60%	74000	5.5	2.6	2.15	1.51	1.45	1.05	0.65
331	25	61%	63000	6.0	2.8	2.10	1.73	1.41	1.27	0.75
326	25	54%	64000	6.1	2.9	2.45	1.72	1.59	1.14	0.67
268	34	46%	46000	7.4	3.5	3.22	2.80	2.35	1.88	1.00
265	42	40%	38000	7.5	3.6	4.40	4.25	3.07	2.93	1.55
243	33	56%	48000	8.1	3.9	3.09	2.56	2.14	1.89	0.96
202	44	50%	36000	9.8	4.6	6.06	4.61	4.57	3.38	1.57
131	51	47%	31000	15.1	7.2	37.86	24.65	18.91	16.17	6.04

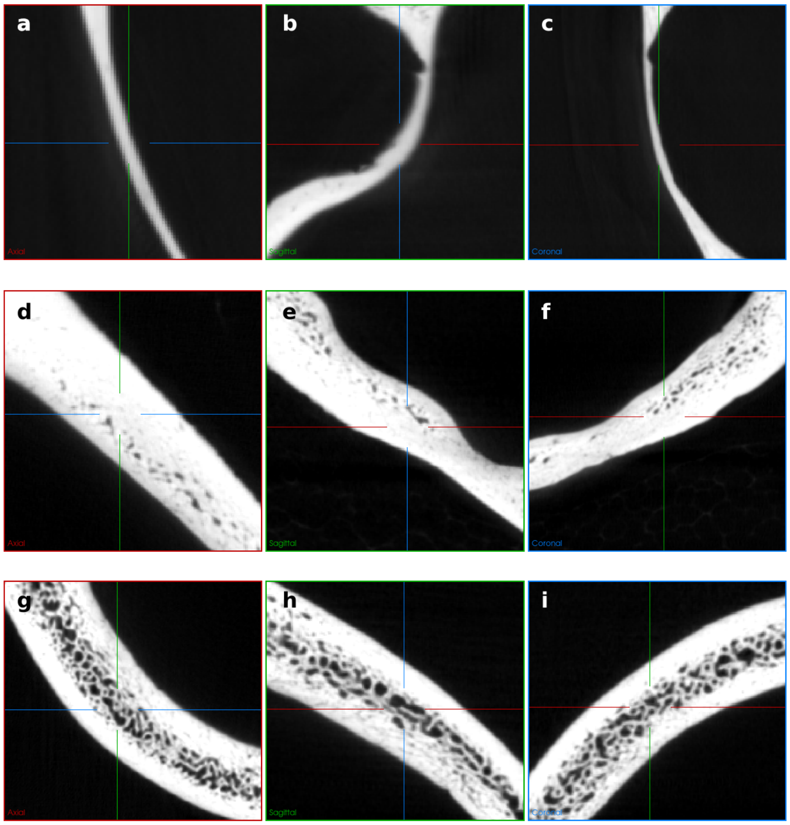

Supplementary Figure 1: Micro-CT slices of the cranial bone measurement locations. **a-c** Axial, sagittal and coronal slices of the frontal cranial bone with thickness of 6–9 mm. **d-f** Axial, sagittal and coronal slices of the occipital cranial bone with thickness of 4–7 mm. **g-i** Axial, sagittal and coronal slices of the temporal cranial bone with thickness of 0.5–1.5 mm.

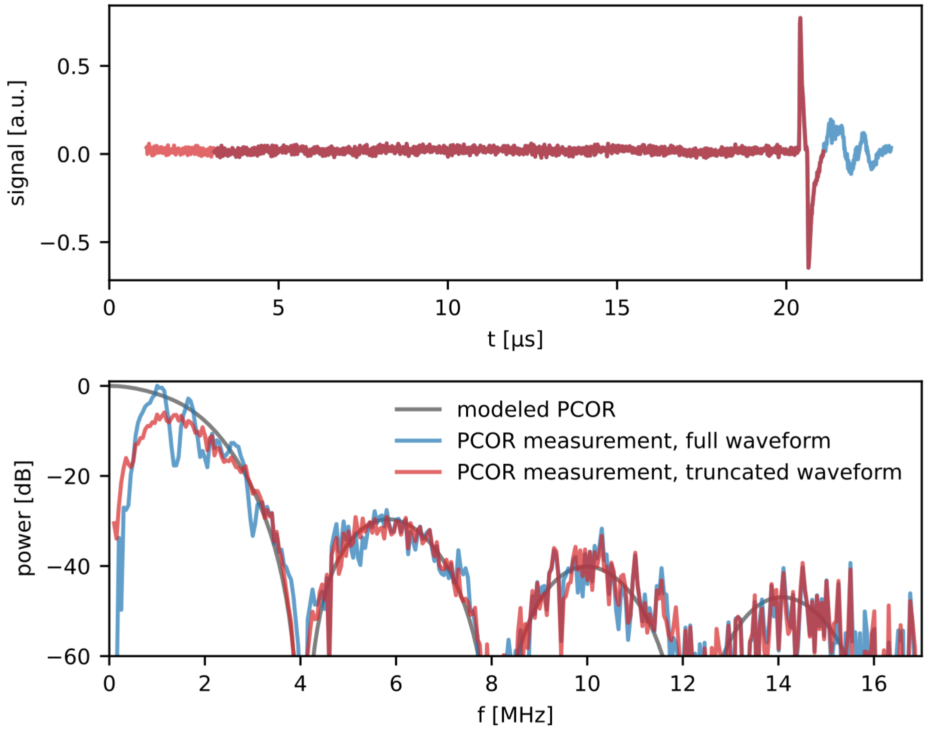

Supplementary Figure 2: Comparison between the simulated and experimental frequency response of a plano-concave optical resonator (PCOR) sensor with a physical thickness of 493 µm. **(top)** A representative experimentally recorded broadband photoacoustic waveform was **(bottom)** Fourier transformed, including internal reflections (blue) and excluding them (red). The acoustic simulation assumes an acoustically planar sensor which causes deviations from the experimental results in dome shaped sensors, especially when the full signal is considered for the fft. Deviations in low frequencies are due to the not-flat acoustic spectrum of the measured PA waveform and due to the data acquisition for the experimental data, which included a 50 kHz first order high-pass filter.

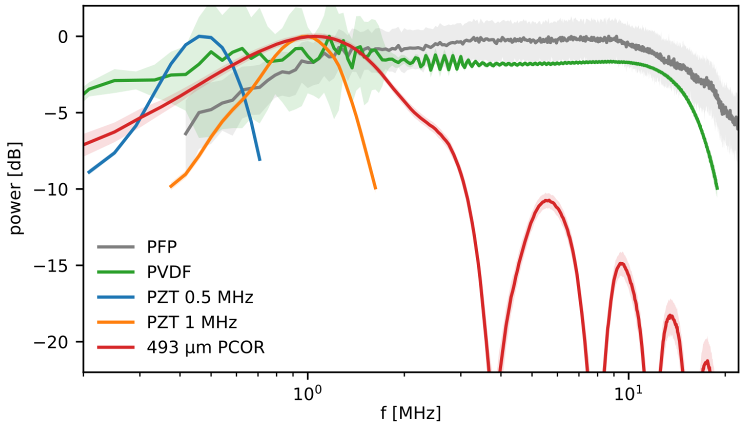

Supplementary Figure 3: Measured acoustic reference spectra of broadband photoacoustic signals acquired using different US sensors. Broadband PA signals were generated in a thin film absorber. It should be noted that the data does not represent the true frequency response of the sensors.
